# Supplementary material for: Local probe-induced structural isomerization in a one-dimensional molecular array
Source: Nat Commun. 2023 Nov 25;14:7741. doi: 10.1038/s41467-023-43659-4 (PMC10676401; doi:10.1038/s41467-023-43659-4)
Supplement: Supplementary file 1 — Supplementary Information [file 41467_2023_43659_MOESM1_ESM.pdf]

Supplementary Information for

Local Probe-induced Structural Isomerization in a One-Dimensional Molecular Array

Shigeki Kawai*[1,2], Orlando J. Silveira[3], Lauri Kurki[3], Zhangyu Yuan[1,2], Tomohiko Nishiuchi[4,5], Takuya Kodama[4,5], Kewei Sun[1], Oscar Custance[1], Jose L. Lado[3], Takashi Kubo*[4,5], Adam S. Foster*[3,6]

[1] *Center for Basic Research on Materials, National Institute for Materials Science, 1-2-1 Sengen, Tsukuba, Ibaraki 305-0047, Japan*

[2] *Graduate School of Pure and Applied Sciences, University of Tsukuba, Tsukuba 305-8571, Japan*

[3] *Department of Applied Physics, Aalto University, 00076 Aalto, Helsinki, Finland*

[4] *Department of Chemistry, Graduate School of Science, Osaka University, Toyonaka, 560-0043, Japan.*

[5] *Innovative Catalysis Science Division (ICS), Institute for Open and Transdisciplinary Research Initiatives (OTRI), Osaka University, Suita, Osaka, 565-0871, Japan.*

[6] *WPI Nano Life Science Institute (WPI-NanoLSI), Kanazawa University, Kakuma- machi, Kanazawa 920-1192, Japan*

Supplementary Note 1-4

Supplementary Figure 1 to 12

Supplementary Table 1

Supplementary Reference List

Supplementary Note 1. NEB calculations of neutral and anionic systems

Supplementary Figure 7 shows the geometry of the fragments of the structure after the debromination used in ORCA. As can be seen in the energy barrier calculated at the PBE level, the relative total energies of the extremes of the reaction 2 → 3 are nearly the same as the ones obtained considering the whole system on a surface, indicating that only the fragments used here are enough to characterize the rearrangement of the rings. Initially, the diradical is around 0.5 eV higher in energy than the dehydroazulene. Upon charging the fragments with one extra electron, no significant changes in the geometry of either the diradical or the dehydroazulene are observed. However, the reaction is inverted, with the dehydroazulene^{-1} nearly 90 meV higher in energy than the diradical^{-1}. The same behavior is observed in the energy barriers calculated with B3LYP, although the diradical has shown to be more stable in relation to the dehydroazulene in comparison with PBE, and the barriers are slightly higher for both neutral and anionic systems.

In a simple picture, without consideration of the full dynamics of the electron, when the molecule is charged e.g. due to interactions with the tip, the diradical stays temporarily energetically favored before the electron transitions into the substrate. Since the barrier is higher for the anionic molecules, they remain in the diradical form as long as the electron stays, supporting the fact that it is possible to observe them in experiments.

Supplementary Note 2. DFT calculations of the diradical and Singlet-triplet energy gap

Single determinant methods such as DFT when applied to open shell systems with two unpaired electrons gives the following broken-symmetry (BS) solution

$$|\psi_{BS}\rangle = |\uparrow\downarrow\rangle. \tag{1}$$

However, the true antiferromagnetic ground state (LS) is the multireference singlet state:

$$|\psi_{LS}\rangle = \frac{1}{\sqrt{2}}(|\uparrow\downarrow\rangle - |\downarrow\uparrow\rangle). \tag{2}$$

And the high-spin (HS) states are the three degenerate triplet states:

$$|\psi^1_{HS}\rangle = \frac{1}{\sqrt{2}}(|\uparrow\downarrow\rangle + |\downarrow\uparrow\rangle), \tag{3}$$

$$|\psi^2_{HS}\rangle = |\uparrow\uparrow\rangle \tag{4}$$

and

$$\left|\psi_{\mathrm{HS}}^{2}\right\rangle = |\downarrow\downarrow\rangle. \tag{5}$$

The renormalized BS solution given by DFT can be written as a linear combination of the singlet and triplet state,

$$|\psi_{\mathrm{BS}}\rangle = \frac{1}{\sqrt{2}}\left(|\psi_{\mathrm{LS}}\rangle + |\psi_{\mathrm{HS}}^{1}\rangle\right), \tag{6}$$

which has the corresponding electronic energy:

$$E_{\mathrm{BS}} = \frac{1}{2}\left(\langle\psi_{\mathrm{LS}}|H|\psi_{\mathrm{LS}}\rangle + \langle\psi_{\mathrm{HS}}^{1}|H|\psi_{\mathrm{HS}}^{1}\rangle\right) = \frac{1}{2}\left(E_{\mathrm{LS}} + E_{\mathrm{HS}}\right). \tag{7}$$

Therefore, the correct singlet ⇒ triplet gap $\Delta E_{\mathrm{ST}} = E_{\mathrm{LS}} - E_{\mathrm{HS}}$ can be written in terms of the total energies of the BS and HS states given by DFT as

$$\Delta E_{\mathrm{ST}} = 2\left(E_{\mathrm{BS}} - E_{\mathrm{HS}}\right). \tag{8}$$

Supplementary Note 3. Molecular orbitals and vibrational modes

In order to study the diradical and its properties in detail, we performed calculations using only a fragment of the ribbon system that stands out from the plane. The dangling bonds left after extracting the fragment were passivated with H atoms, and only this portion was enough already to reproduce the spin density of the complete system as well as the singlet-triplet energy gap with the same functional. Molecular orbitals, energy levels and vibrational modes were obtained using the B3LYP functional, which includes a fraction of exact exchange to the DFT exchange-correlation functional in order to improve the description of the electronic properties. Selected molecular orbitals and energy levels are shown in Supplementary Figure 10. In Tab. 1 is shown the frequencies of some vibrational modes of the diradical fragment. Not all vibrational modes can be used to represent the true system, since only a fragment was used. A tag named “relevant” was then attributed to each node, with “yes” meaning that only atoms far from the passivated parts of the fragment participate effectively in the specific vibration. Another tag was attributed to each mode concerning the symmetry of the vibration in the plane of the diradical fragment. “Sym” means that the mode is symmetric in relation to the center of the molecule, whereas “ant” means that the mode is antisymmetric and “out” means that the mode has out-of-plane components. To illustrate some relevant modes, four selected modes are shown in Supplementary Figure 12, where the arrows indicate the direction of movement of each atom.

Supplementary Note 4. XXZ model

The feature at ±150 meV in the dI/dV signal could also be attributed to further spin-splitting excitations due to anisotropy in the system. We then have made use of the Python library dmrgpy[S1] to compute the eigenvalues of the Heisenberg Hamiltonian with the anisotropic term $(J+J_z)S_1^zS_2^z$. The top panel of Supplementary Figure 11 shows the evolution of the splitting as J_z changes in relation to J, until the $S_1^zS_2^z$ vanishes completely. The bottom panel of Supplementary Figure 11 shows the simulated dI/dV spectra for selected values of J_z revealing that the second step could be associated with another spin-spliting excitation. We stress here, however, that the anisotropy associated to the C atoms is less than 1% of J =100 meV, whereas around 70% of J would be necessary to reproduce the experimental data, further confirming that the step is in fact associated with vibrational excitations.

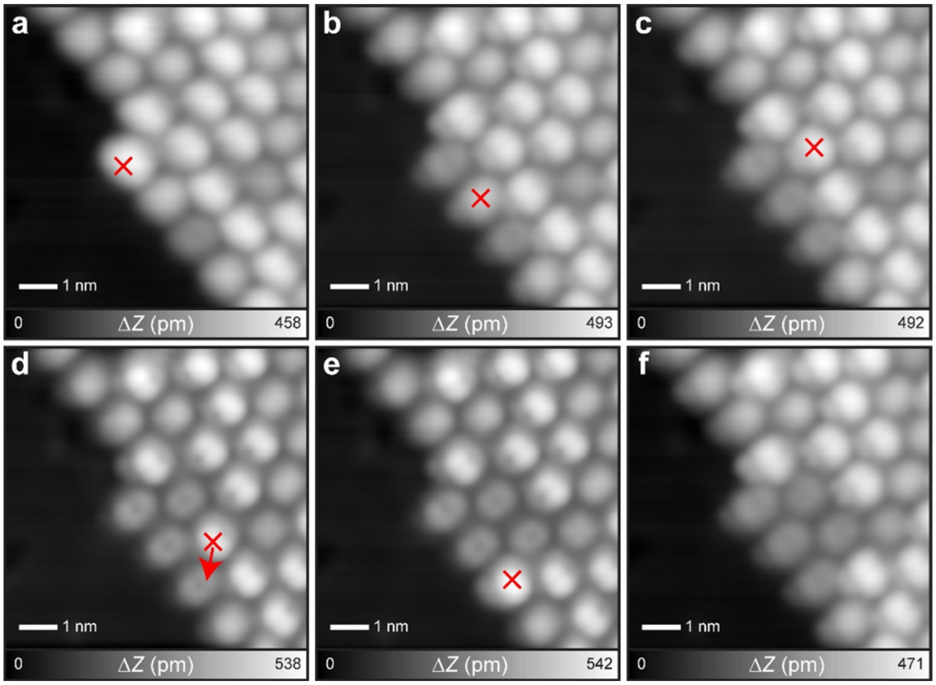

Supplementary Figure 1. A series of tip-induced debromination on 3D-OMC on Ag(111). (a)-(f) The tip-induced debromination sites are indicated by crosses. The debromination process induces repositioning of the Br atom to the adjacent radical site as indicated by an arrow. Measurement parameters: $V = 1.0$ V and $I = 5$ pA.

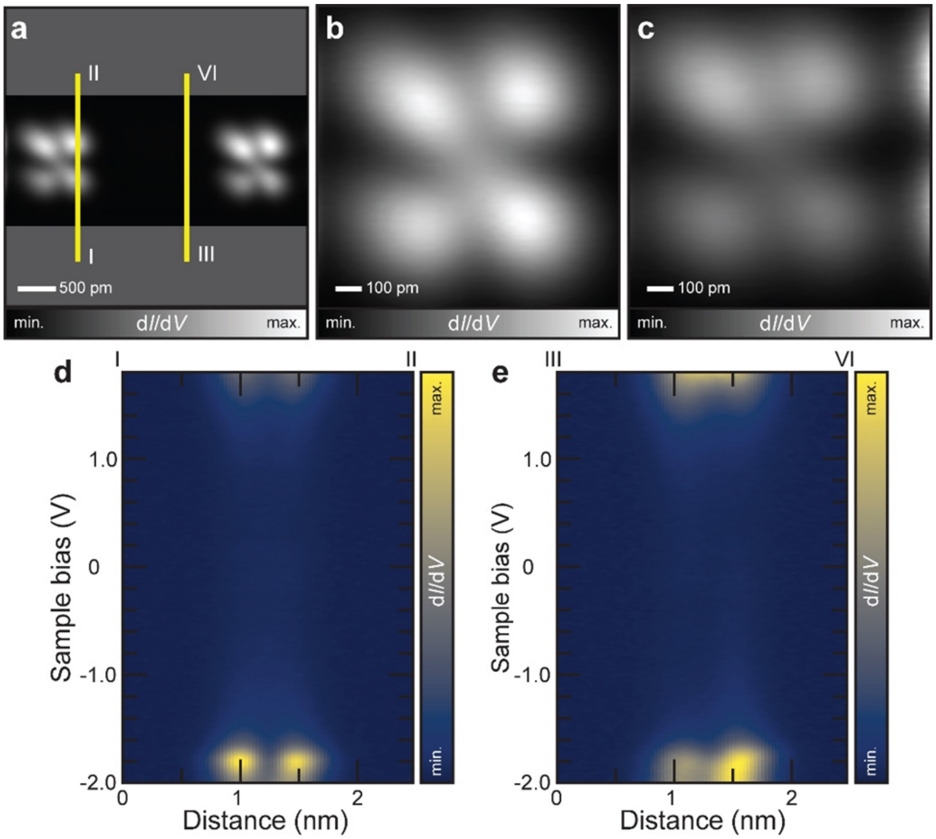

Supplementary Figure 2. **Electric properties of the units before and after debromination. (a)** Constant-height d*I*/d*V* map of the intact and fully-debrominated units on Ag(111) and **(b,c)** their close views. Note that the tip was set closet to the surface to **(d,e)** Two-dimensional d*I*/d*V* maps of the intact and fully-debrominated units, taken along I-II and III-VI lines, respectively. Measurement parameters: V= 1.5 V in (A), V=-1.8 V in (b,c). V_{ac} = 10mV and f = 512 Hz in (a-e).

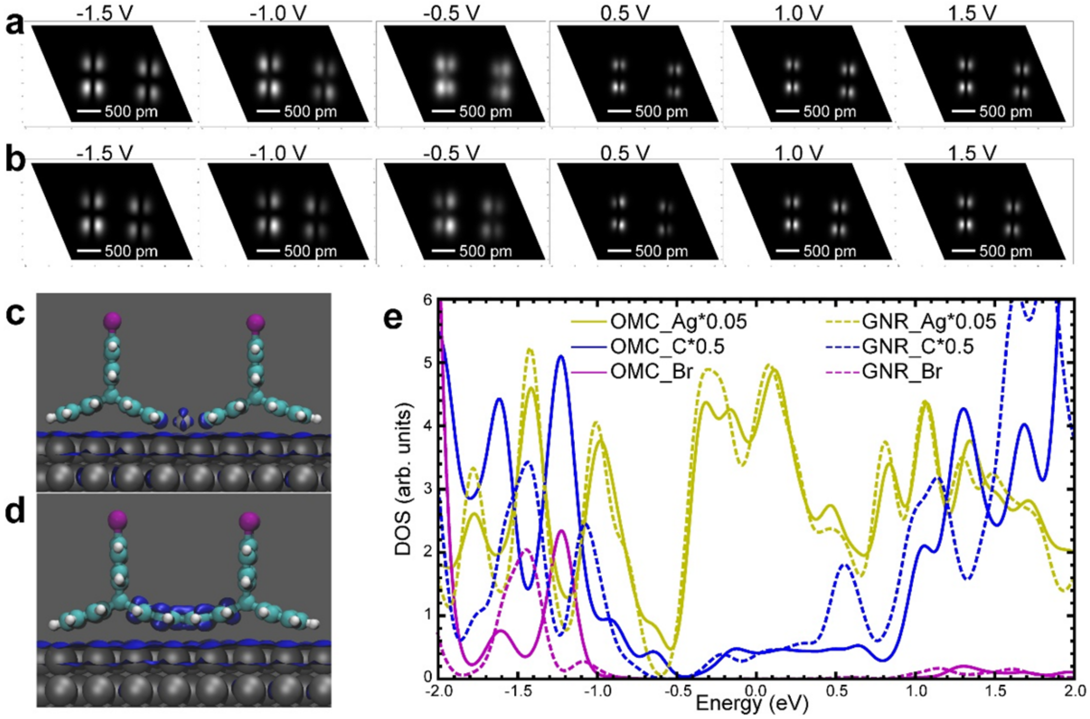

Supplementary Figure 3. Comparison of the calculated properties of OMC and GNR model molecular structures adsorbed on the Ag(111) substrate. Constant height simulated STM images for (**a**) OMC and (**b**) GNR. Charge density isocontour at 0.16 eÅ^{-3} associated with gap states (-0.2 to 0.6 eV) for (**c**) OMC and (**d**) GNR, as shown in the (**e**) Density of States plot. Purple, green, white, and gray balls in (c,d) correspond to Br, C, H, and Ag atoms.

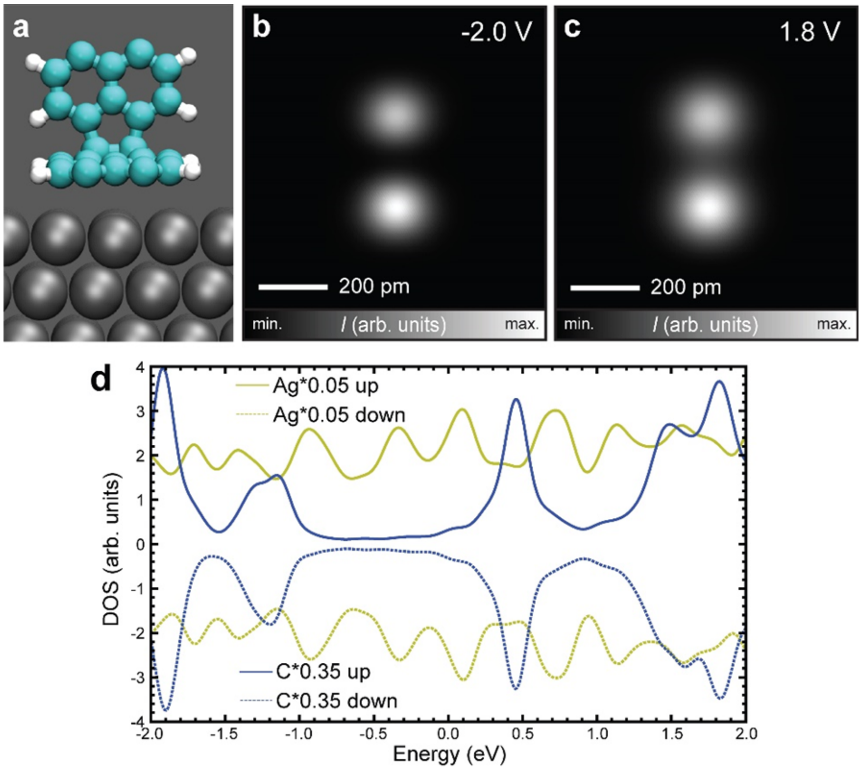

Supplementary Figure 4. Calculated properties of the diradical on the Ag(111) surface. (**a**) Atomic structure. Predicted constant height STM images at bias voltages of (**b**) -1.8 V and (**c**) +1.8 V. (**d**) Projected Density of States.

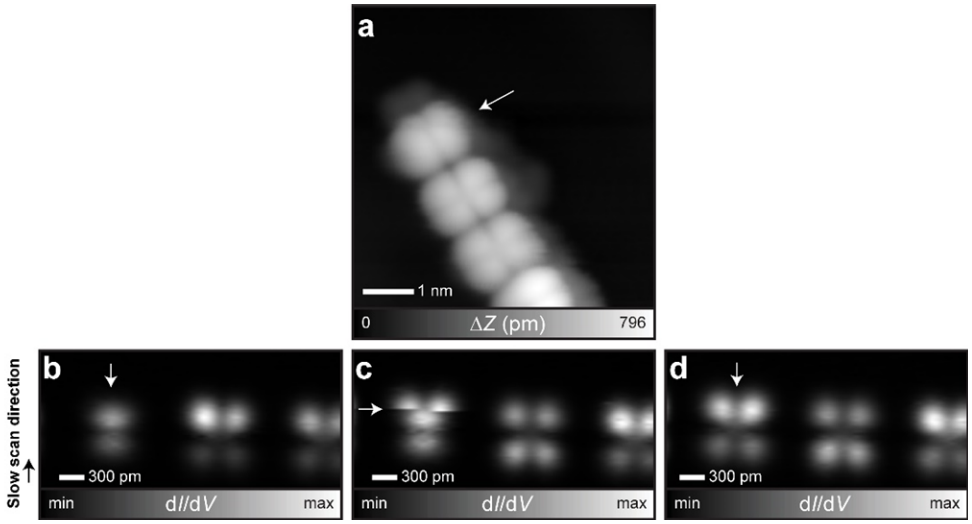

Supplementary Figure 5. Infrequent change of the diradical unit. (a) STM topography of the fully-debrominated units in the 3D-OMC formed on Au(111). The contrast of the unit at the terminus differed from those of other units. **(b-d)** A series of constant height d*I*/d*V* maps measured with a bias voltage of -1.75 V at different heights. The tip-sample distance in C is closer than that in B by 50 pm and further than that in D by 60 pm. The contrast of the fully-debrominated unit at the terminus was switched to the one, which is the same as the other. Once switched, no reverse switch was caused even upon setting the tip closer to the molecule. Since the contrast of the unit indicated by an arrow in B is the same as that of diradical obtained with DFT calculations in Supplementary Figure 4, the unit corresponds to the diradical species. Measurement parameters: V = -2.0 V and I = 5 pA in (a) and V = -1.75 V in (b-d). V_{ac} = 10mV and f = 512 Hz in (b-d).

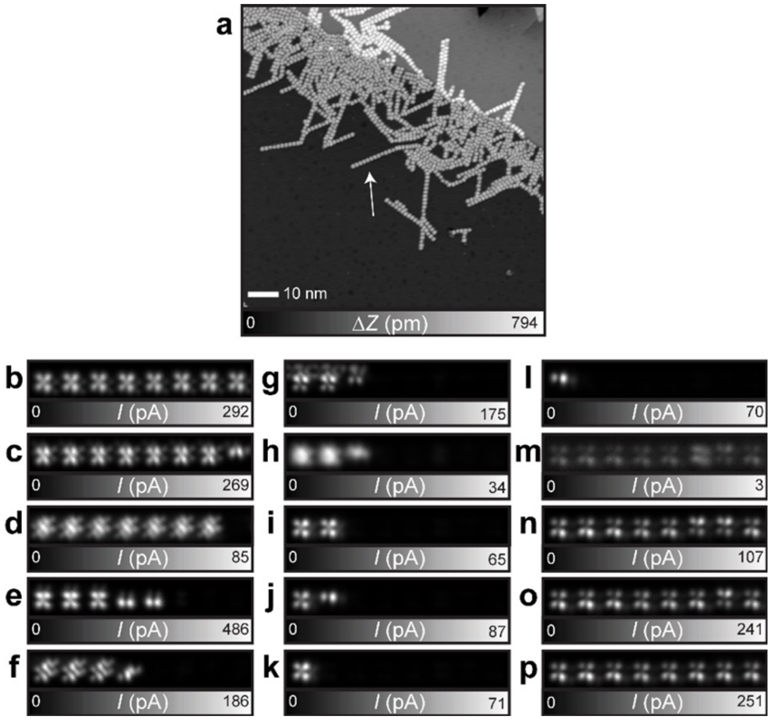

Supplementary Figure 6. Preparation of the breadboard for 8-bit ascii code. (**a**) Large-scale STM topography of a 3D-OMC formed on Ag(111). (**b-l**) A series of constant-height current maps taken during the tip-induced debromination and (**m-p**) the tip-induced isomerization. The image sizes of (b-p) are 12.1 nm × 2.2 nm. The code was set to 0000 0000 in (p). Measurement parameters: V= -2.0 V.

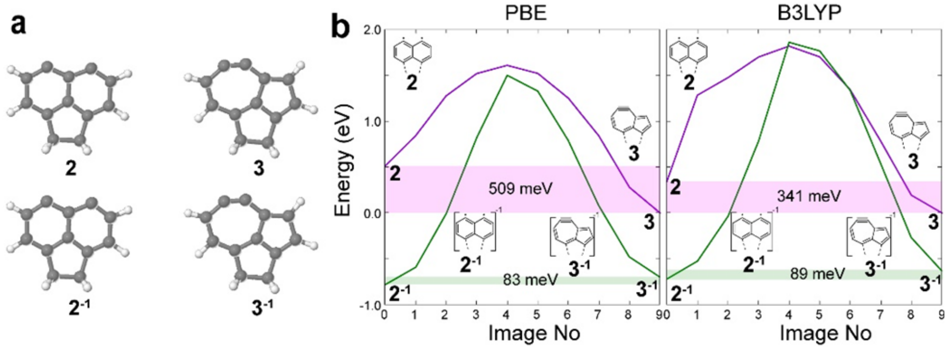

Supplementary Figure 7. (**a**) Geometry of the neutral and anionic fragments relaxed at the B3LYP level. (**b**) Energy barriers of the reactions **2** → **3** and $\mathbf{3^{-1}}$ → $\mathbf{2^{-1}}$ calculated at two different levels PBE and B3LYP.

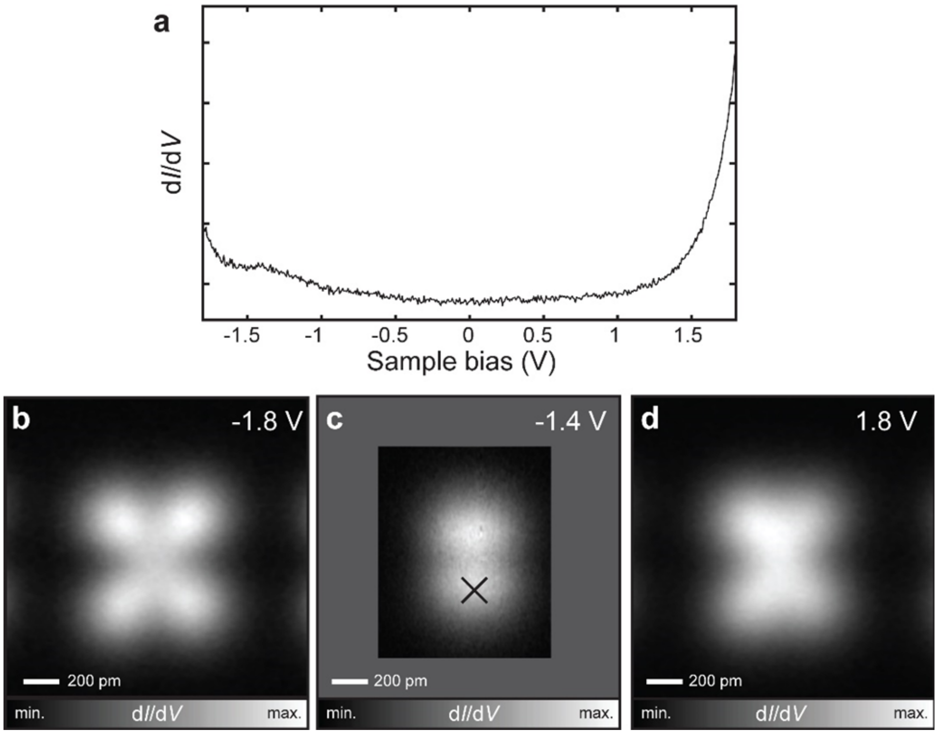

Supplementary Figure 8. Electronic properties of the diradical unit. (a) d*I*/d*V* measured. (**b**) d*I*/d*V* maps measured at -1.8 B, (**c**) at -1.4 V, and (**d**) 1.8 V. Measurement parameters, V_{ac} = 10 mV in (a) and V_{ac} = 5mV in (b-d), and *f* = 512 Hz.

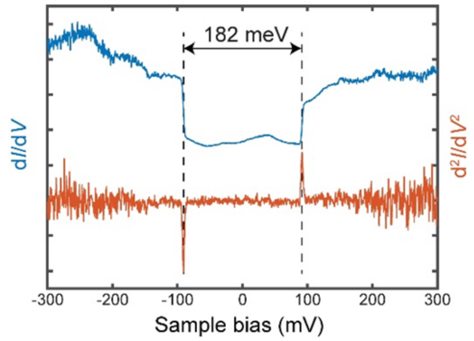

Supplementary Figure 9. Scanning tunneling spectroscopy of the diradical unit. Differential conductance and second derivative curves. Measured parameters: V_{ac}= 2 mV and *f* = 512 Hz.

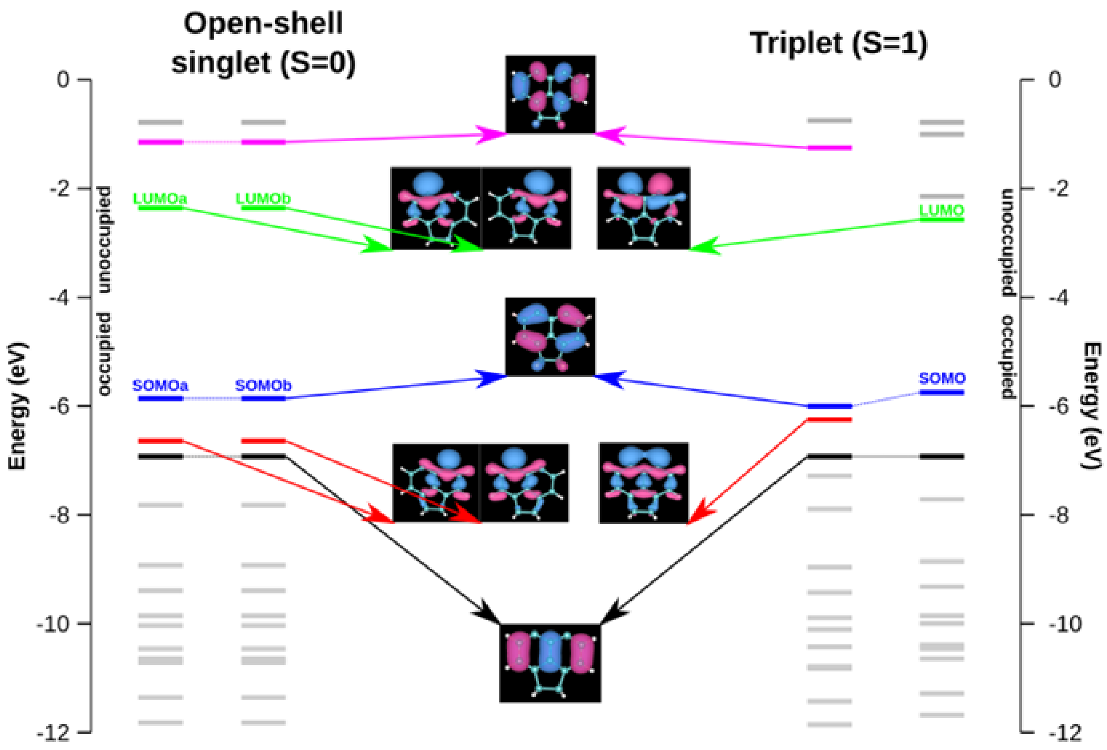

Supplementary Figure 10. Molecular orbitals of the singlet and triplet states of the diradical structure. Green and white balls correspond to C and H atoms.

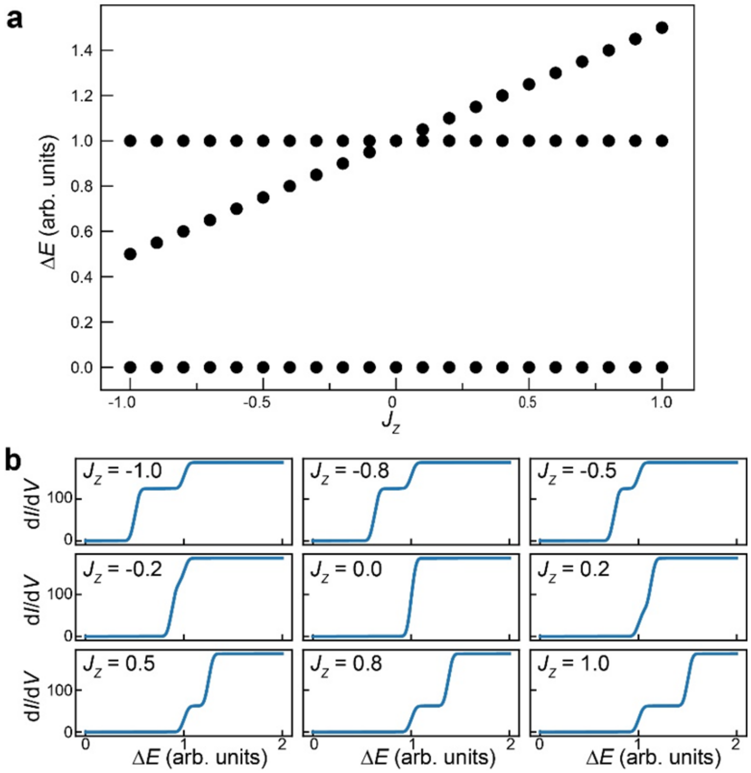

Supplementary Figure 11. Evolution of the splitting as the anisotropic term J_z . (a) Evolution of the energy levels and (**b**) dI/dV simulations of the XXZ model in respect to the anisotropic term J_z. In the calculations, we considered J =1.

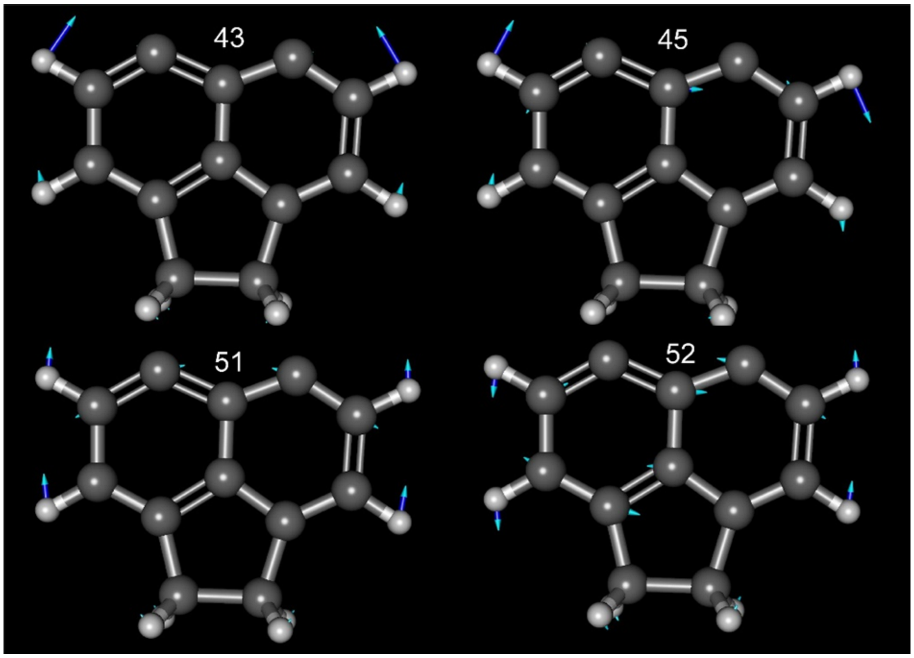

Supplementary Figure 12. Example of relevant vibrational modes. The arrows indicate the displacement of the atoms in the vibrational mode. The numbers correspond to the nodes of the vibration indicated in Supplementary Table 1.

Supplementary Table 1: Vibrational frequencies of the fragment of the diradical structure. Only frequencies above 100 meV and below 300 meV are shown in the table. The cases with a gray background have their displacements shown in Supplementary Figure 12.

node	freq (cm^{-1})	freq (meV)	relevant	sym/ant/out	node	freq (cm^{-1})	freq (meV)	relevant	sym/ant/out
26	847.5	101.7	no	-	40	1245.91	149.5092	no	-
27	909.48	109.1376	yes	out	41	1298.13	155.7756	yes	ant
28	923.14	110.7768	yes	out	42	1308.85	157.062	yes	sym
29	948.28	113.7936	no	-	43	1349.79	161.9748	yes	sym
30	980.25	117.63	yes	sym	44	1373.54	164.8248	yes	sym
31	1021.1	122.532	no	-	45	1431.43	171.7716	yes	ant
32	1029.93	123.5916	yes	ant	46	1446.09	173.5308	no	-
33	1081.03	129.7236	yes	sym	47	1467.55	176.106	no	-
34	1111.05	133.326	yes	ant	48	1495.18	179.4216	no	-
35	1171.56	140.5872	no	-	49	1495.88	179.5056	no	-
36	1198.24	143.7888	yes	sym	50	1557.38	186.8856	yes	sym
37	1200.39	144.0468	yes	ant	51	1634.5	196.14	yes	sym
38	1228.33	147.3996	yes	ant	52	1638.88	196.6656	yes	ant
39	1244.4	149.328	no	-					

Supplementary reference

1. Lado, J. L. DMRGPy. https://github.com/joselado/dmrgpy (2023)
