# Supplementary material for: Local probe-induced structural isomerization in a one-dimensional molecular array
Source: Nat Commun. 2023 Nov 25;14:7741. doi: 10.1038/s41467-023-43659-4 (PMC10676401; doi:10.1038/s41467-023-43659-4)
Supplement: Supplementary file 3 — Description of Additional Supplementary Files [file 41467_2023_43659_MOESM3_ESM.pdf]

Description of Additional Supplementary Files

File Name: Supplementary Movie 1
Description: Two structures of the dehydroazulene isomer units were used to embed the text following binary ASCII code.

File Name: Supplementary Movie 2
Description: Three structures of the dehydroazulene isomers and diradical units were u used to embed the text following ternary ASCII code.
